# Encephalitic rabies following cat bites: two cases in northern Uganda

**DOI:** 10.1136/pn-2026-005099

**Published:** 2026-04-11

**Authors:** Dan Langoya Oriba, Annet Adongpiny, Neil Scolding

**Affiliations:** 1 Medicine Department, St Mary's Hospital Lacor, Gulu, Uganda; 2 Internal Medicine Department, Gulu University, Gulu, Northern Region, Uganda; 3 Medicine Department, Alebtong Health Centre IV, Alebtong, Uganda; 4 Department of Neurology, Gloucestershire Hospitals NHS Foundation Trust, Gloucester, UK

**Keywords:** NEUROVIROLOGY

## Abstract

Although rabies continues to exert a major global health impact, only a very small minority of UK neurologists will ever have encountered cases, and many remain unfamiliar with its diagnosis and management. The recent death of a UK national, in Yorkshire, emphasises the importance of general neurologists having familiarity with the manifestations of rabies. Here, we describe two cases of Ugandan males who developed rapidly fatal encephalitic rabies following cat bites. We outline their clinical features and summarise current diagnostic and management approaches.

## Introduction

 Novel forms of encephalitic illness continue to be described—related to channel antibodies,^1^ COVID-19 (both virus and vaccines),^2 3^ and new therapeutic agents for malignancies,^4^ to take just three instances—and occupy much space in the neurological literature and in training curricula. However, long-known encephalitic illnesses continue to exert a major global health impact, and yet often remain totally unfamiliar to practising neurological clinicians. For example, only a very small minority of UK neurologists will ever have encountered a case of one of the most serious and dramatic of the encephalitides, rabies. The sad recent death of a UK national, diagnosed in Yorkshire and the Humber,^5^ emphasises the need for all of us to have more than passing familiarity with the manifestations of rabies.

Rabies is remarkable for perhaps three particularly unsettling features: it is one of the very few viruses that can infect all mammals^6 7^; it carries a mortality rate of virtually 100%^7–10^; and while its incubation period typically lies between 21 and 90 days, in some 10% of the cases it is over a year^9^—indeed, there are well-documented cases presenting 4–6 years after exposure,^11 12^ with some reports of an incubation period of well over a decade.^7 13–15^

Rabies encephalitis is caused by the neurotropic virus of the family Rhabdoviridae, genus Lyssavirus, subgroup rabies virus (figure 1). It is transmitted via saliva that contaminates bites, scratches, wounds and any other form of recent mucosal exposure. Globally, 99% of human cases follow bites from dogs. Other culprit reservoir mammals include foxes, coyotes, cats, raccoons, skunks and bats.^8^ Here, we present from northern Uganda two cases of African males admitted as emergencies to the same hospital, who both developed rapidly fatal encephalitic rabies following cat bites.

**Figure 1:**
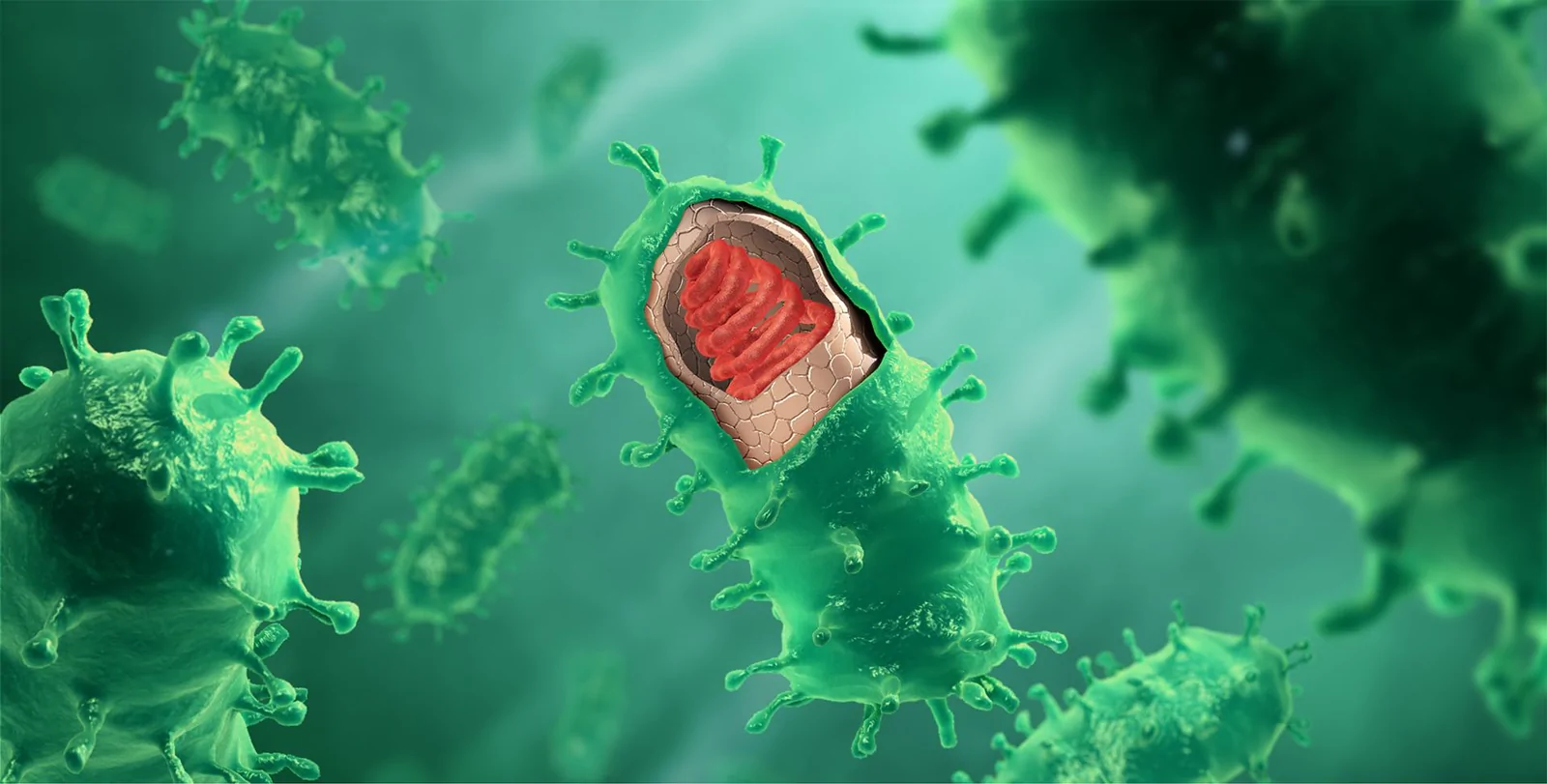
Rabies virus. www.scientificanimations.com/wiki-images/ -http://www.scientificanimations.com/wiki-images/ CC BY-SA 4.0

## Case presentations

### Case 1

A man in his late teens with no prior chronic illness presented to the Emergency Department at St Mary’s Hospital, Lacor, northern Uganda (figure 2) with confusion and restlessness. He had severe odynophagia and also a fever for the previous 48 hours. There was no history of voice change, cough, dyspnoea, loss of consciousness or convulsions, and no suggestion of alcohol or illicit drug use. His social history was significant for engaging in hunting activities principally in the local bush and forest. In recent days, he had, while hunting, encountered two cats fighting in a village; he had intervened and sustained a trivial but skin-penetrating cat bite. (The interval between presentation and cat bite was unclear: the patient himself was confused, and different carers gave varying accounts of when the event occurred.)

**Figure 2:**
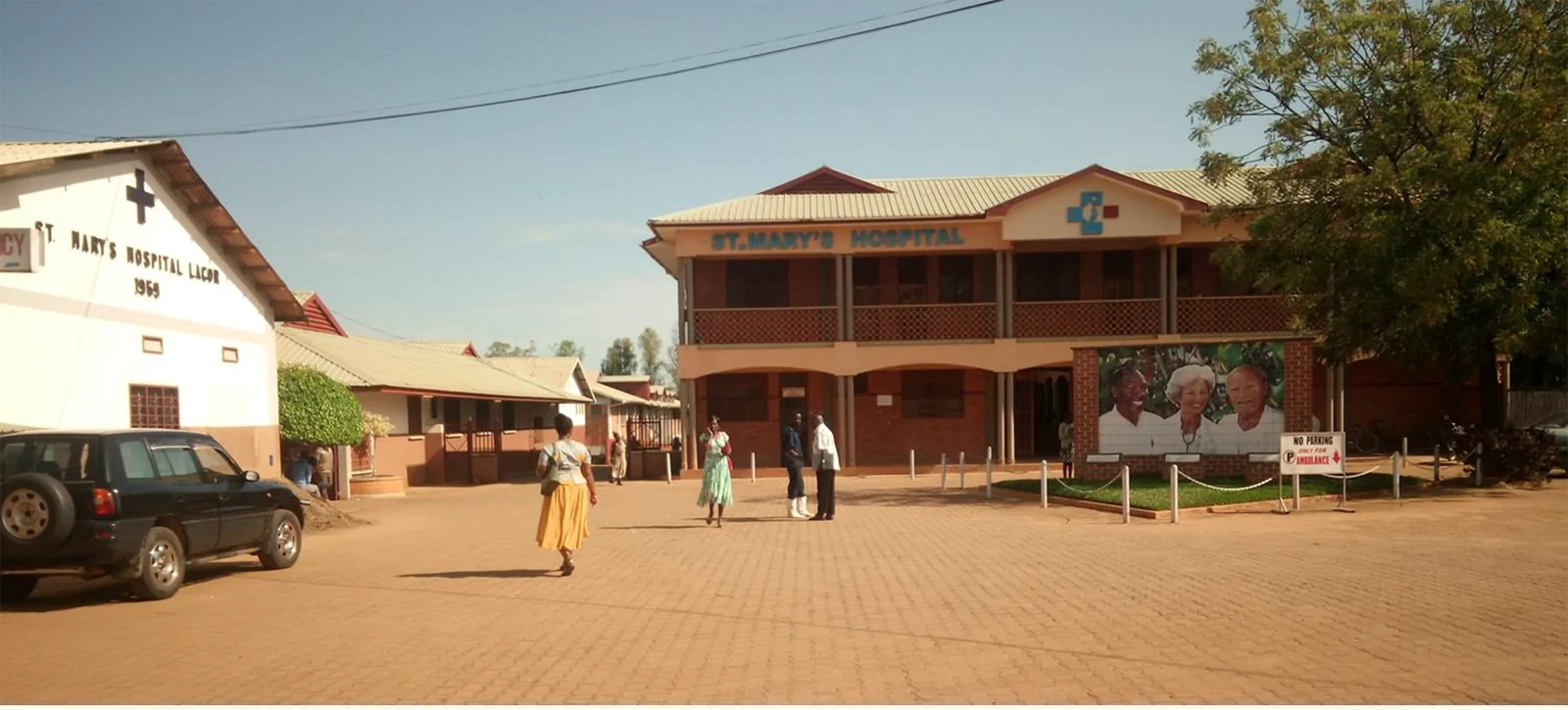
St Mary’s Hospital, Lacor, northern Uganda.

On examination, he was confused, with episodes of severe psychomotor agitation—careering around the ward incoherently screaming, extremely distressed and apparently terrified. Marked hydrophobia was apparent. He had features of autonomic dysfunction including drooling of saliva and persistently elevated blood pressures. Local examination of the pharynx was normal, and aside from a tachycardia and hypertension, other body systems were unremarkable.

A full blood count showed mild leucocytosis with neutrophilia, and a blood film was negative for malaria parasites.

Clinically, the impression was that he showed the classical features of encephalitic rabies. The patient was isolated and managed supportively with opioid analgesics (intramuscular tramadol), maintenance intravenous fluids (Ringer’s lactate), and anxiolytics—intravenous diazepam followed later by intramuscular chlorpromazine. However, his encephalitic features progressively worsened; he rapidly developed coma and died 15 hours after admission; resuscitation was not attempted.

### Case 2

A man in his late 20s sustained a bite from a wild cat during a visit to Kitgum District, Northern Uganda. The wounds were superficially cleaned at home. He did not seek medical care; he did not receive post-exposure vaccination or rabies immunoglobulin at any point.

Approximately 3 months later, he began visiting local health facilities complaining of persistent itching at the bite site and burning sensations radiating along the affected limb. His wife reported a notable change in his behaviour, with markedly heightened sexual desire. Despite multiple consultations, no suspicion of rabies was raised, and no clinician recommended vaccination.

Some 6 months post-exposure, the patient was rushed to the emergency unit with profuse sweating, hypersalivation, acute urinary retention and severe dysuria. He was initially managed empirically for suspected urosepsis. However, within hours of admission, his condition deteriorated dramatically with the development of pronounced hydrophobia, aggressive behaviour and agitation, rapidly worsening confusion and escalating autonomic instability with blood pressure rising to 171/102 mmHg and a tachycardia of 130–157 bpm. A clinical diagnosis of encephalitic rabies was apparent. Supportive management was instituted; the patient died within 24 hours of admission.

## Discussion

While rare in the UK, rabies continues to cause 60–70 000 deaths globally each year, the great majority in Africa (around 35%) and Asia (around 60%).^6 16^ Following contact with (and usually trauma from) a rabid animal, the virus multiplies locally. On encountering a peripheral nerve terminal, the rabies virus binds to acetylcholine receptors and thereby gains neuronal cell entry. It then travels centrally, mainly through retrograde axoplasmic flow. Eventually, continued viral replication within the central nervous system leads to inflammation and neuronal death, manifesting as progressive encephalitis and death. Dorsal root ganglia cell death and inflammation may contribute to pain, itching or paraesthesia at the site of entry, while anterograde spread of virus results in infection of various tissues—including in particular the salivary glands, causing the classic sialorrhoea. Bites to the face and hands—both densely innervated areas—increase the risk of clinical disease, the former often with shorter incubation periods as a consequence of shorter nerve lengths.

In the early clinical stages, there are only non-specific viral symptoms, although injury site paraesthesiae and/or pruritus may offer early hints of rabies. Neurological disease takes the form of ‘furious’ rabies in some 80% of patients, paralytic (‘dumb’) rabies in 20%. The reasons for this dichotomy are unknown, though furious disease is thought to result from predominant involvement of the limbic system, paralytic rabies from spinal anterior horn infection.^17^ Paralysis may also occur in the late stages of furious rabies. In furious rabies, confusion and severe agitation are accompanied by hydrophobia, with often dramatic pharyngeal muscle spasms triggered by seeing, hearing or—remarkably—just thinking of water. Objectively—if examination is possible—meningism may be present, with generalised hyper-reflexia and extensor plantar responses. Coma and death commonly follow within a few days (at most), although with intensive care, survival can be over a month.

In dumb rabies, there is an ascending, progressive paralysis, usually starting in the bitten limb. Ultimately, the respiratory musculature becomes involved. Fever and confusion are often present, sometimes seizures: the diagnosis is usually far less obvious, the differential wider. Autonomic dysfunction can occur (in both forms) and indeed, there may be direct cardiac involvement with features of myocarditis.

A confident clinical diagnosis is usually feasible, as in these cases. In resource-rich settings, immunological or molecular tests on four types of samples—serum, saliva, cerebrospinal fluid and a nuchal skin biopsy—can confirm the diagnosis.^18^ The Rapid Fluorescent Focus Inhibition Test (RFFIT) can identify rabies virus antibodies in serum or CSF; immunofluorescence testing can also show rabies antigen in nuchal skin biopsies. RT-PCR may disclose rabies RNA in saliva, CSF or serum samples, or in skin biopsies or (post-mortem) brain tissue. None of these was available readily in our hospital setting.

Unfamiliarity with rabies is by no means limited to resource-rich settings. Despite its lethality, rabies is still classified by the WHO as a neglected tropical disease^19^ that is under-appreciated and under-recognised by healthcare practitioners in many endemic areas, as well-illustrated by Case 2 above. Lack of rapid diagnostic tools and limited public health reporting compounds the problem. Furthermore, preventive strategies that are the main area of focus with documented high efficacy are unfortunately neither affordable nor accessible to many victims in resource-constrained settings.

The recent UK case proves it is by no means ‘luxury knowledge’ for UK-based neurologists to be familiar with the clinical recognition of rabies (box 1), with the basic principles of management, including anti-rabies vaccine protocols for post-exposure prophylaxis, along with vigorous wound debridement and cleaning (iodine or alcohol) and, where appropriate, direct administration of human rabies immune globulin into the wound. The case triggered the release of new UK rabies management guidelines in June 2025.^20–23^ Appropriate management depends on the ‘composite rabies risk’,^21^ determined following assessment of the category of exposure—principally considering the wound severity and contact with saliva, the country of origin and animal species involved^22^ to determine the post-exposure treatment required (figure 3).^23^ Post-exposure prophylaxis is said to be 100% effective if instituted within 24 hours of exposure, and if properly executed. These cases highlight, however, the need for awareness and vigilance. Cat-bite rabies is extremely uncommon, though by no means unreported.^24 25^

Box 1 **When to think about rabies?**

**Red flags for rabies**

1. Encephalitis of unclear cause (applies to ‘dumb’ rabies in particular; furious rabies less likely to be missed)
  - Any patient with acute encephalitis where no alternative diagnosis is found, especially if there is any history of animal exposure, even if minor or not recent
  - Encephalitis with progressive paralysis (again, ‘dumb’ rabies)
  - Rapid progression and a CSF pleocytosis should raise suspicion
2. Neurological features that are the classic rabies ‘red flag’ signs:
  - Hydrophobia (involuntary pharyngeal spasms triggered by attempts to drink)
  - Aerophobia (airflow over the face triggering spasms)
  - Autonomic instability (tachycardia, hypersalivation, sweating)
  - Agitation, confusion, hallucinations, delirium, insomnia and seizures are all relatively common in other encephalitides, but should at least raise the possibility of rabies
3. Local symptoms at the exposure site
  - Paraesthesia, pain, itching or dysaesthesia at the bite/scratch site—a particularly important early clue.

**Figure 3:**
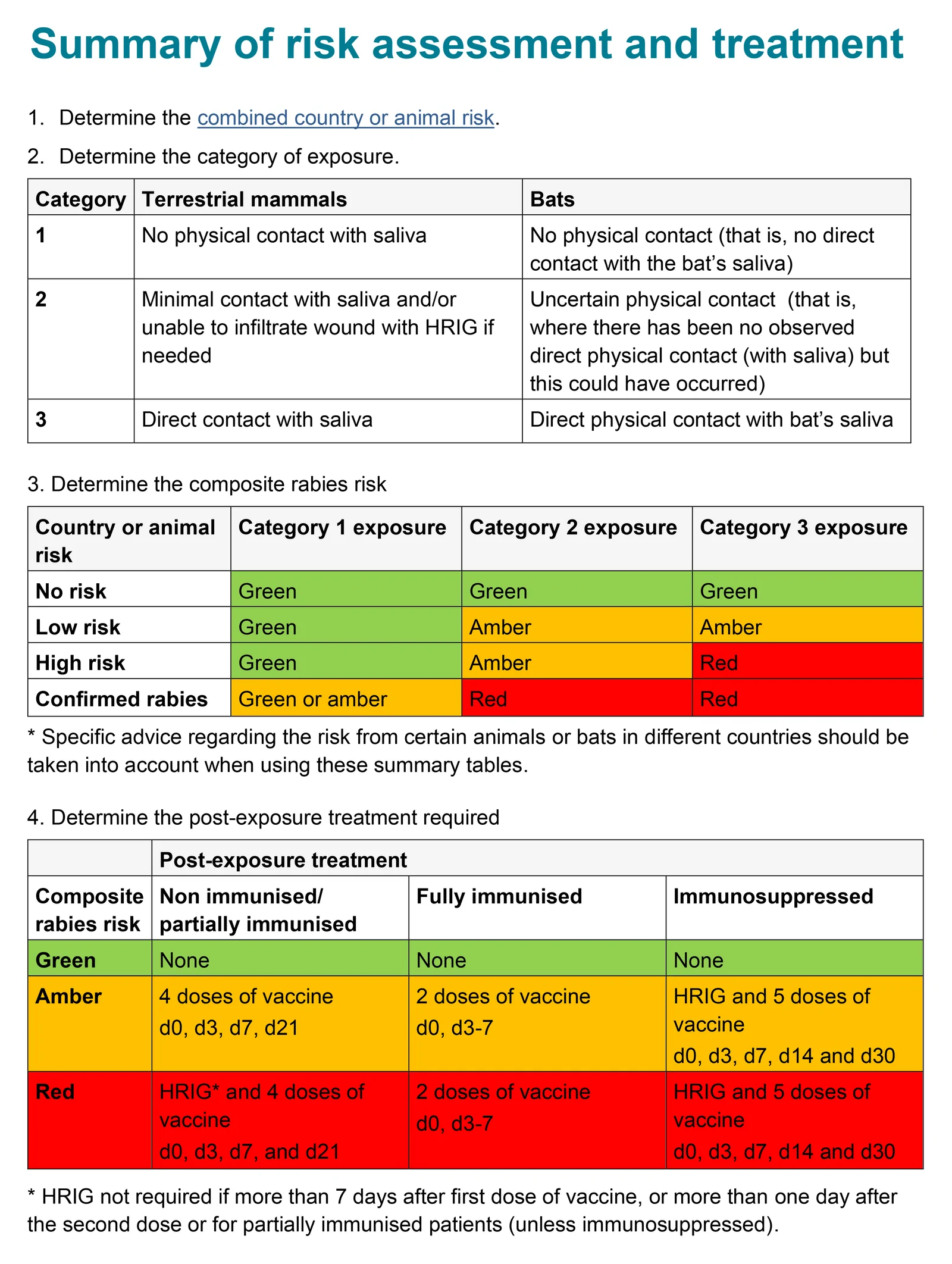
UK Health Security agency recommendations for post-exposure rabies management: risk assessment and treatment.^23^ HRIG, human rabies immunoglobulin.

**Key points**

- The death in Sheffield from rabies in 2025 of a Yorkshire woman emphasises the importance for neurologists of knowing the manifestations and management of rabies, even where it is non-endemic.
- Rabies is remarkable for carrying a mortality rate of virtually 100%.
- Its typical incubation period is between 21 and 90 days, but in 10% of the cases, it is over a year.

**Further reading**

- Fooks, A. R. *et al*. Rabies. *Nat Rev Dis Primers* 2017;3:17091. https://doi.org/10.1038/nrdp.2017.91
- UK Health Security Agency: Rabies: summary of risk assessment and treatment (2025); Rabies post-exposure treatment: management guidelines (2025), and Rabies risks in terrestrial animals by country (2025), all available at https://www.gov.uk/health-and-social-care/health-protection-infectious-diseases
- Gadre G, Satishchandra P, Mahadevan A, *et al*. Rabies viral encephalitis: clinical determinants in diagnosis with special reference to paralytic form. *J Neurol Neurosurg Psychiatry* 2010;81:812–20. 10.1136/jnnp.2009.185504

## Data Availability

No data are available.
